# The prevalence and incidence of glaucoma in Norway 2004–2018: A nationwide population-based study

**DOI:** 10.1371/journal.pone.0242786

**Published:** 2020-12-10

**Authors:** Jon Klokk Slettedal, Valgerður Dóra Traustadóttir, Leiv Sandvik, Amund Ringvold

**Affiliations:** 1 Department of Ophthalmology, Oslo University Hospital, Oslo, Norway; 2 Institute of Clinical Medicine, University of Oslo, Oslo, Norway; 3 Oslo Center for Biostatistics and Epidemiology, Oslo University Hospital, Oslo, Norway; Oregon Health and Science University, UNITED STATES

## Abstract

**Purpose:**

To describe prevalence, life-time prevalence and incidence of glaucoma in Norway over a 15-year period.

**Materials and methods:**

Data from The Norwegian Prescription Database was used to identify all prescriptions for glaucoma medication during the period 2004 to 2018. Population figures and lifespan data were obtained from The National Bureau of Statistics.

**Results:**

Of a population of 5.3 million, a total of 75733 patients using glaucoma eye drops were identified in 2018. The national prevalence was thus 1.4%, whilst in those over 70 years of age, 8.0%. When divided into counties, the prevalence varied between 1.1 and 1.9%. Overall, the prevalence was stable in the period 2004–2018. Life time prevalence was found to be 9.4% for men and 10.2% for women. National one-year incidence proportion per 10000 was 17.0 for the total population and a peak incidence of 93.8/10000 in the 80–89 year age group was identified.

**Conclusions:**

Glaucoma prevalence remained stable during the period 2004–2018, while incidence decreased slightly in the elderly population.

## Introduction

Glaucoma is the leading cause of irreversible blindness in developed countries. It most commonly starts with a prolonged asymptomatic phase with increasing atrophy of the optic nerve, and eventually, without proper treatment, leading to a progressive and irreversible loss of vision [1]. Most patients have an elevated intraocular pressure. The disease is often difficult to detect in the early stages and the diagnosis of glaucoma requires, in most cases, an examination by a trained ophthalmologist. This makes it challenging to establish the exact prevalence and incidence of glaucoma. Furthermore, there are geographical variations in the types of glaucoma and the exact prevalence of these types [2].

The word glaucoma roots back to the ancient Greek word «glaukos», which means blue, green, or gray, and the generic term was coined centuries before the various subtypes of the disease were described. Today the following distinct entities have been identified: primary open-angle glaucoma (POAG), primary angle closure glaucoma (PACG), secondary glaucoma including pseudoexfoliation glaucoma (PEG), and congenital/juvenile glaucoma. In Europe the most common glaucoma types are POAG and PEG, together largely making up the group open angle glaucoma (OAG).

Glaucoma is a multifactorial disease linked to a variety of etiological factors. Due to similarities in both symptomatology, therapy, and outcome of the various subtypes, it is logical to analyze glaucoma as a group without specifying subunits. Given that the population is aging, and as glaucoma prevalence increases with age, estimates on glaucoma prevalence and incidence are highly relevant because glaucoma is likely to pose a significant burden on society in the years to come.

On a global basis, glaucoma prevalence, through meta-analysis, for a population aged 40–80 years has recently been estimated to be 3.5%, with the trend significantly increasing [2]. However, there is a fundamental problem as to how to identify glaucomatous damage, and thus there is marked variation in inclusion criteria in various cohorts. This figure is likely to hide marked regional variations, with both over- and underdiagnosis of the disease likely [3, 4]. In addition, it has been shown that figures for OAG vary considerably according to racial and geographical differences [5–9]. Comparison is, however, difficult because sample size and age distribution differ between reports. Cohort-based studies from the 5 Nordic countries show marked differences in glaucoma prevalence both between regions and countries [10].

A Danish study estimated glaucoma prevalence in the Nordic countries from the total consumption of anti-glaucomatous drugs in a defined area [11]. Another report based on data from the national prescription agency during the period 1996–2011 found increasing prevalence in Denmark, and concluded that glaucoma is likely to impose an increasing public health challenge in the years to come [12].

Incidence studies on OAG are rather few, and the reported rates show remarkable deviations, from 0.943 to 9.0 per 1000 person-years [13–15].

A nationwide prevalence and incidence study is lacking in Norway, although the information is much needed for Medicare reasons, future planning of research projects and public health demands. The present report aims to meet these requirements.

## Material and methods

The present study is based on data obtained from The Norwegian Prescription Database during the period 2004–2018. Every resident of Norway has a unique identification number. This personal number is used for all prescriptions. Glaucoma medication is covered by the “blue prescription system” and is, excluding a minimal annual charge, provided free of charge to patients.

Given that a prescription is mandatory for all glaucoma drugs, we can assume that the prevalence of glaucoma is closely estimated by the number of patients claiming medication with a prescription. Furthermore, topical IOP-lowering drugs have no other use, and there is essentially no abuse of these drugs (with the possible exception of using prostaglandin analogues cosmetically for growing long and dark eyelashes). No new classes of drugs have been introduced in the study period.

A person, identified through their unique national identification number, was included if they claimed anti-glaucomatous topical medication with a prescription at a pharmacy between 2004 and 2018. Medication was identified using ATC-codes (Anatomical Therapeutic Chemical Classification system) starting with S01E, excluding S01EC01 (acetazolamide for peroral use). Prevalence was found by dividing all persons claiming glaucoma medication with the population (at 1^st^ of July of a given year) in that area and/or age group. If an individual moved to another county, this patient was registered in the county where he or she claimed the first prescription that year. No person was registered in two different counties in the same year.

One-year incidence proportions was found by identifying all new persons claiming anti-glaucomatous topical medication during one year who did not claim any such drugs during the previous year. This number of new cases was then divided by the population at risk; i.e. the population in that area and/or age group subtracting the number of persons with glaucoma at the start of the year. A prescription was necessary to obtain glaucoma medication and such a prescription was given with multiple supplies of medication for 3 months use. The prescription was valid for one year after issue, thus the patients being treated for glaucoma had to claim medication several times during a year at a pharmacy. Glaucoma is a non-lethal disease and once diagnosed, persons are not cured.

In order to illustrate the lower and upper limits of prevalence of glaucoma and trends over time, we have calculated these values for all counties in the period from 2004 to 2018 and included the values for the county with lowest prevalence and the county with highest prevalence in 2018 in the charts for both prevalence and incidence proportions.

We obtained data for both genders, in the following age groups: 0–49 years, 50–59, 60–69, 70–79, 80–89, and 90 years and older. In addition to national numbers, we included data for all 18 counties of Norway in order to examine for geographical variation. In order to calculate p-value when comparing prevalence or incidence in two groups, a chi-square test was used. This statistical calculation was performed by using SPSS 23.

Lifespan data from The National Bureau of Statistics was obtained to calculate life-time prevalence of glaucoma. The following formula was used: Life-time prevalence = a1*p1 + a2*p2 + a3*p3 + a4*p4 + a5*p5 + a6*p6, where a1, a2, a3, a4, a5, a6 denote proportion of persons with life expectancy in the age groups 0–49, 50–59, 60–69, 70–79, 80–89, and 90+ years and p1, p2, p3, p4, p5, p6 are the glaucoma prevalence in the same age groups.

Ethical approval was not required by The regional committee for medical and health research ethics, given this was a register-based study.

## Results

### Prevalence

**Fig 1:**
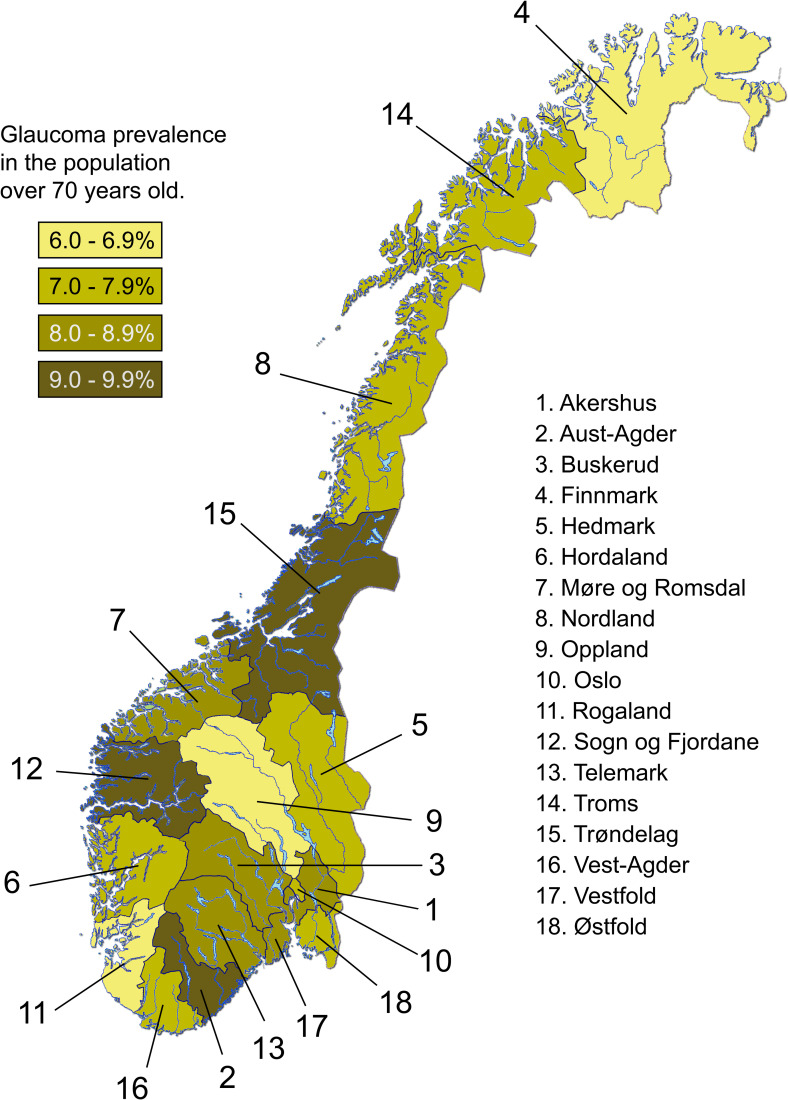
Glaucoma prevalence by Norwegian county for the age group over 70 years.

In total, 75733 patients were treated with IOP-lowering eyedrops in Norway in 2018. The national, age irrespective, glaucoma prevalence in Norway in 2018 was 1.4% (men 1.3%, women 1.6%), while for those over 50 years of age it was 3.7% (men 3.5%, women 4.0%), and for those 70 years and over, 8.0% (men 7.6% and women 8.6% respectively) (Table 1). Table 1 also shows age-adjusted prevalence figures divided by county, whereas the geographical distribution is illustrated in Fig 1.

**Table 1:** Glaucoma prevalence in Norway and in 18 counties, 2018.

Area	Inhabitants 2018	Prevalence, total (per 1000)	Prevalence, women (per 1000)	Prevalence, men (per 1000)	Prevalence over 70 years old, both genders (per 1000)
**Norway**	**5311800**	**14.3**	**15.6**	**13.0**	**80.0**
Akershus	619043	14.6	16.2	13.1	89.0
Aust-Agder	117440	18.4	19.5	17.4	98.2
Buskerud	282454	15.0	16.6	13.4	80.8
Finnmark	76024	13.9	16.1	11.8	68.2
Hedmark	197157	16.8	17.5	16.1	77.0
Hordaland	523562	14.1	15.4	12.8	79.0
Møre og Romsdal	267118	15.1	16.8	13.4	80.1
Nordland	243324	15.7	17.5	13.9	74.0
Oppland	189710	13.3	13.8	12.7	62.8
Oslo	677269	10.6	11.9	9.4	79.9
Rogaland	474611	10.5	11.1	9.9	64.7
Sogn og Fjordane	109985	19.3	20.9	17.8	94.2
Telemark	173345	16.8	18.0	15.7	82.1
Troms	166855	13.6	14.8	12.3	70.6
Trøndelag	460328	17.4	19.0	15.8	96.4
Vest-Agder	187044	12.7	14.0	11.4	74.0
Vestfold	250060	16.4	18.1	14.8	84.4
Østfold	296471	14.0	15.6	12.5	73.2

**Fig 2:**
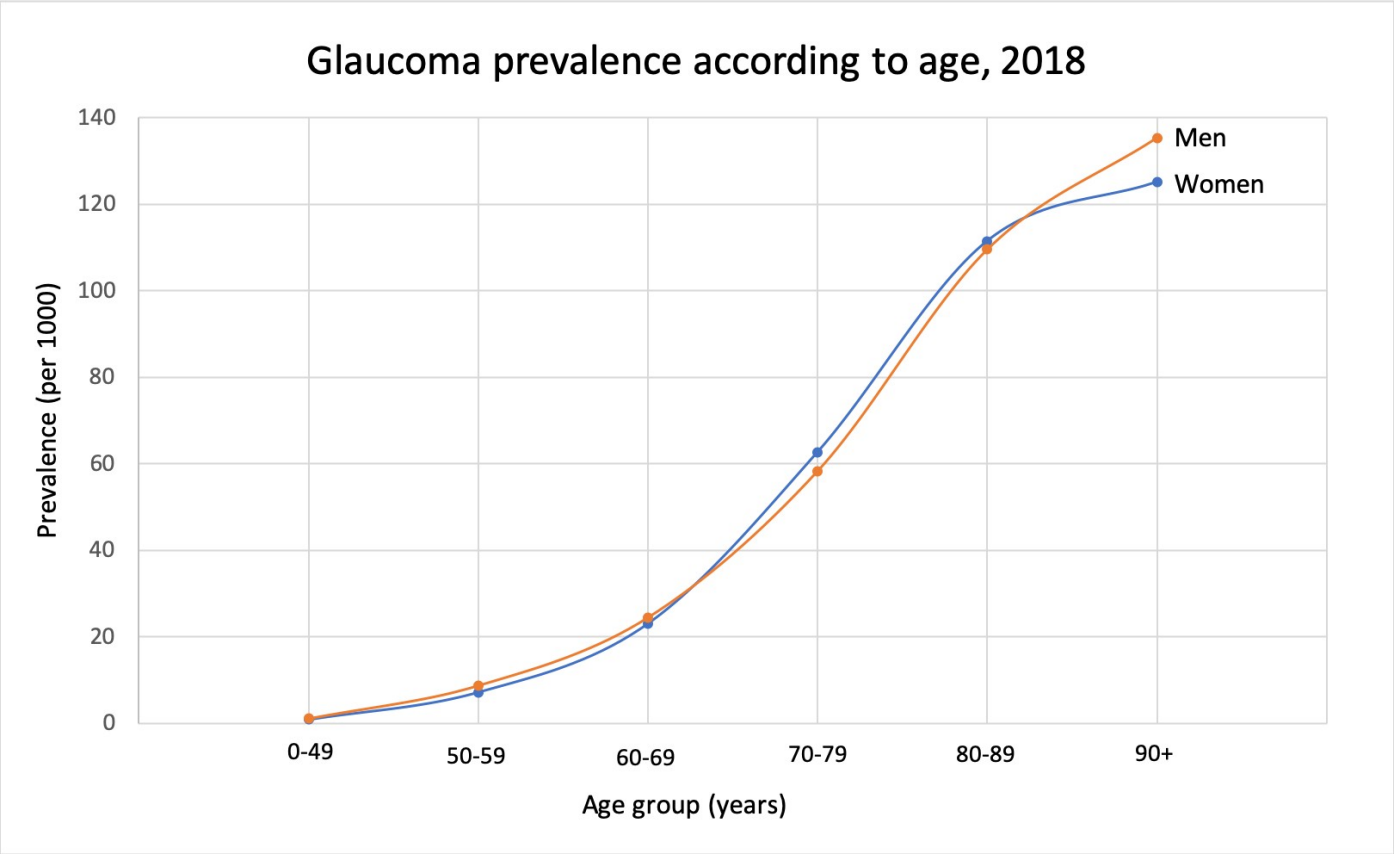
Glaucoma prevalence by sex in different age groups in Norway 2018.

The glaucoma prevalence according to age was similar in each age group for both genders, except for the age group 90+ where the prevalence was slightly and significantly higher for males than for females (1.33% vs 1.24%, p = 0.012, Fig 2). Lifetime prevalence of glaucoma in the total population of Norway was estimated to be 9.4% for men and 10.2% for women (p<0.001).

**Fig 3:**
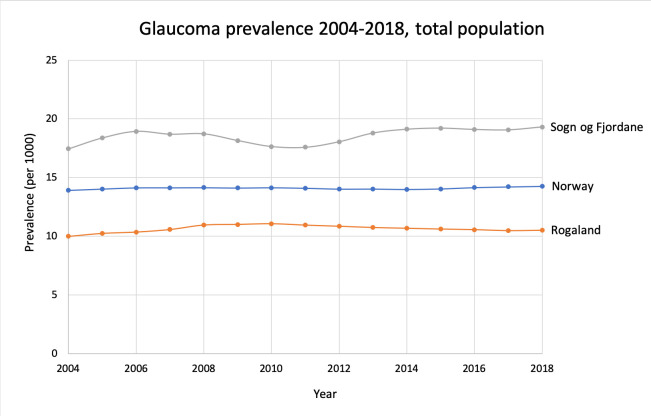
Glaucoma prevalence 2004–2018 for Norway, and the counties with the highest and lowest prevalence in 2018.

The number of patients treated for glaucoma with eye drops rose from 63862 in 2004 to 75733 in 2018. However, as the population increased in the same period, the prevalence showed only a slight, but significant increase from 1.39% to 1.43% (p<0.001, Fig 3).

The highest and lowest prevalence was found in the counties Sogn og Fjordane (1.93%) and Rogaland (1.05%), respectively. Some variations occurred on a county-basis during the observation period, whereas the national figures remained stable (Fig 3).

**Fig 4:**
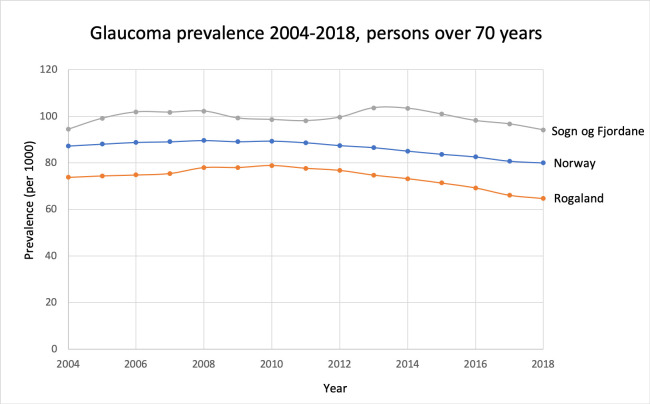
Glaucoma prevalence proportions 2004–2018 for the age group 70 years and older for Norway and the counties with highest and lowest prevalence in the total population in 2018.

Comparable prevalence for persons aged 70 years and over are seen in Fig 4. The national prevalence in this group was 8.7% in 2004, peaking in 2008 at 9.0%, and thereafter gradually reducing to 8.0% in 2018. The prevalence decreased slightly and significantly in the elderly population (p<0.001, Fig 4).

### Incidence

**Fig 5:**
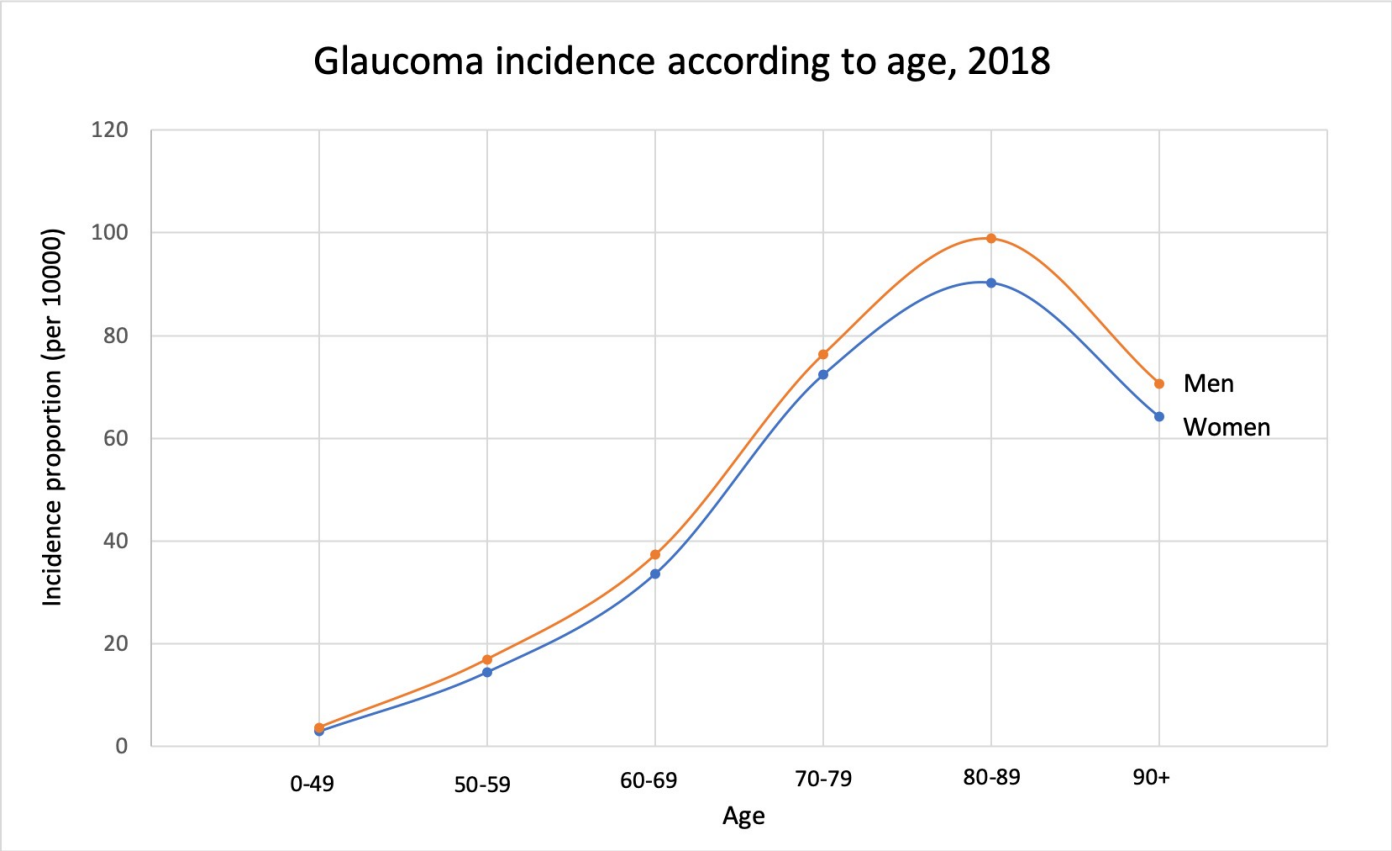
Glaucoma incidence proportions by sex in different age groups in Norway 2018.

**Fig 6:**
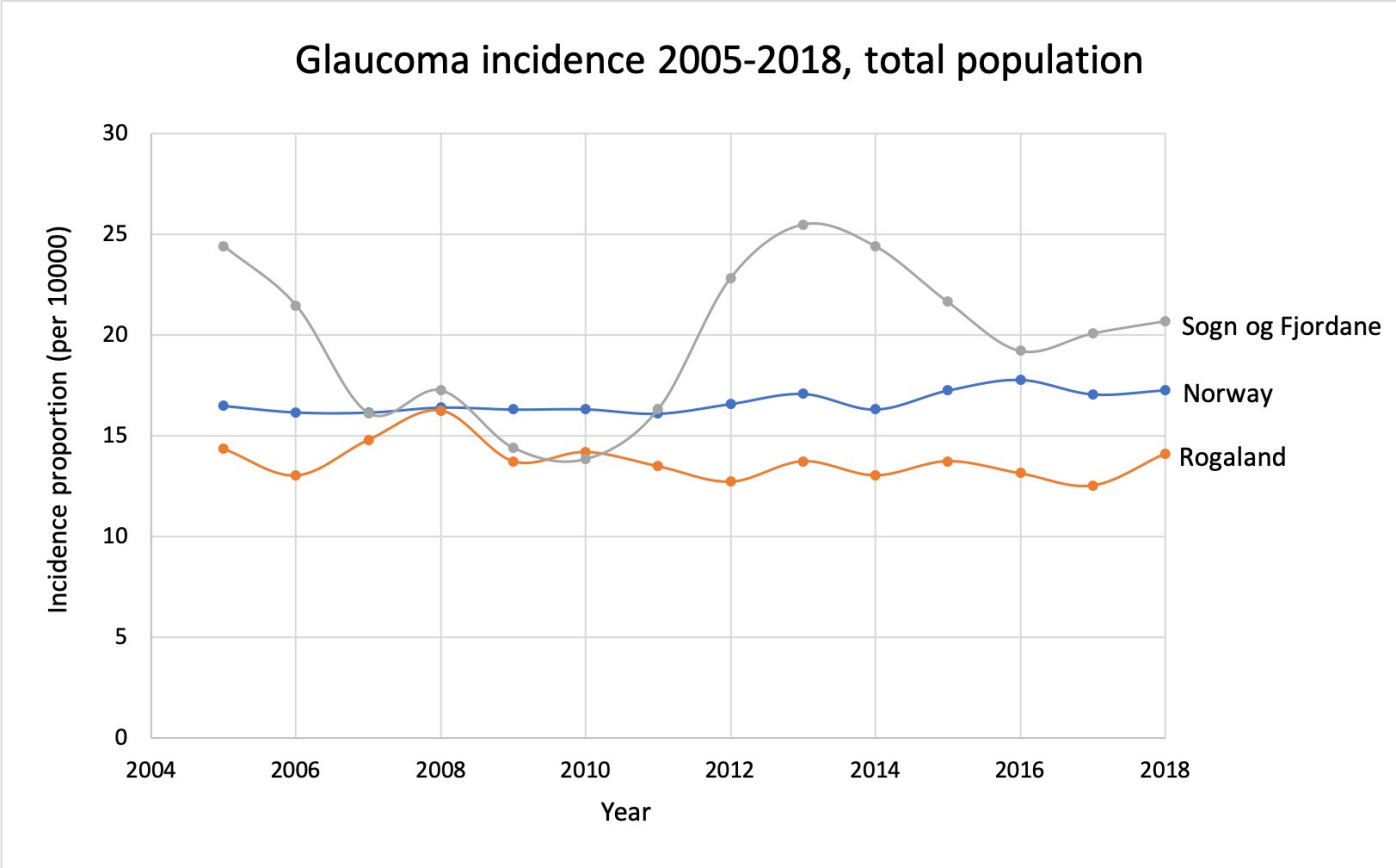
Glaucoma incidence proportions 2005–2018 for Norway and the counties with highest and lowest prevalence in 2018.

**Fig 7:**
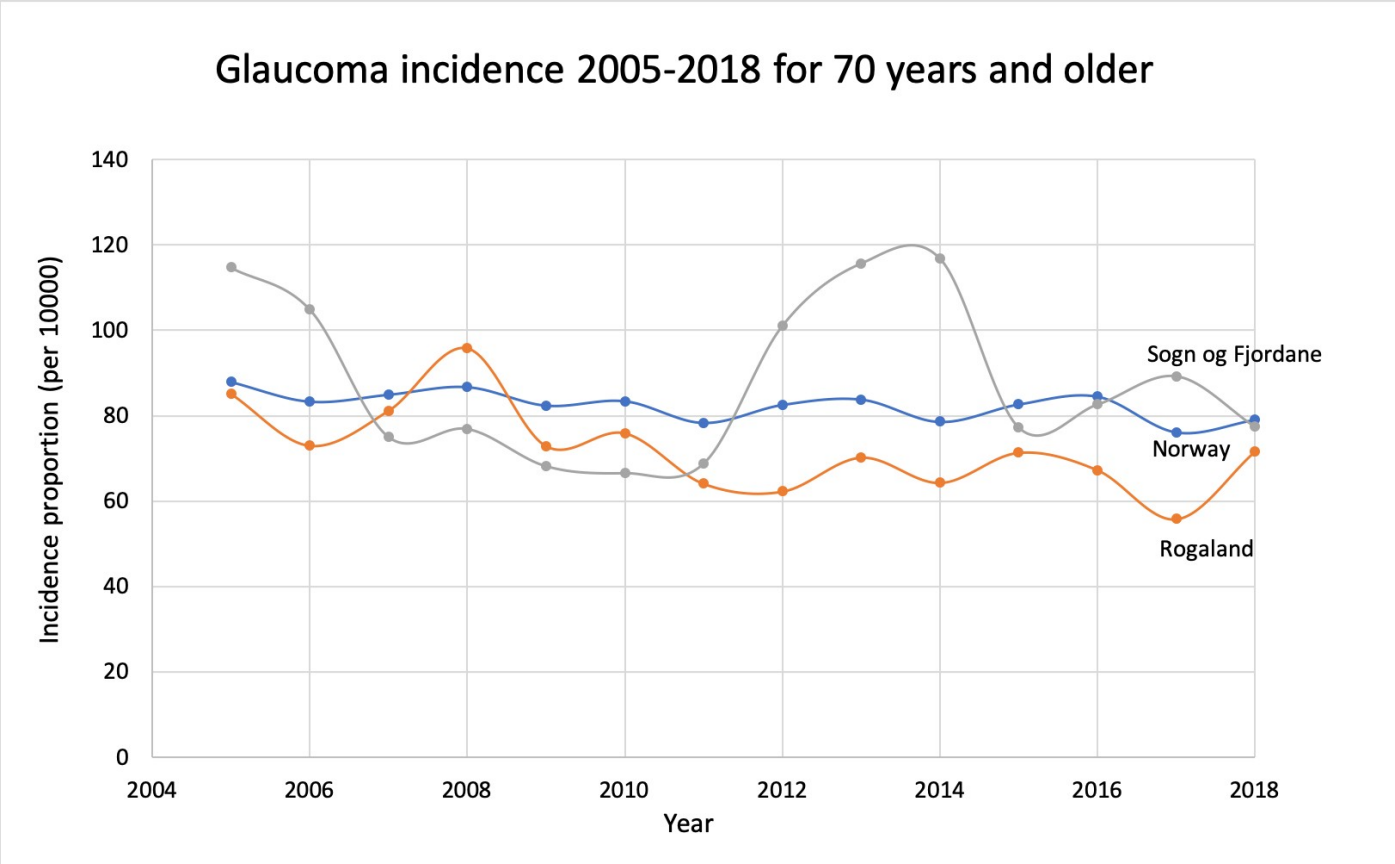
Glaucoma incidence proportions 2005–2018 for the age group 70 years and older for Norway and the counties with highest and lowest prevalence in 2018.

The same set-up for calculations was used as in the prevalence section (Table 2). In 2018 there were 9034 (4537 men, 4497 women) new patients treated for glaucoma. The incidence proportion in different age groups is illustrated in Fig 5. One-year incidence trends during the period 2005–2018 are shown in Figs 6 and 7. The incidence proportion of glaucoma was slightly higher in males than females in each age group above 50 years. This difference was significant in the age groups 70–79, 80–89, and 90+ (p<0.01).

**Table 2:** Glaucoma incidence proportions in Norway and in 18 counties, 2018.

Area	Inhabitants without glaucoma 2018	Incidence proportions, total (per 1000)	Incidence proportions, women (per 1000)	Incidence proportions, men (per 1000)	Incidence proportions, over 70 years old, both genders (per 1000)
**Norway**	**5236064**	**1.7**	**1.7**	**1.7**	**7.9**
Akershus	609988	1.6	1.7	1.6	8.5
Aust-Agder	115274	1.8	1.6	1.9	7.1
Buskerud	278208	1.7	1.8	1.6	8.0
Finnmark	74968	2.2	2.4	2.1	8.8
Hedmark	193851	2.1	2.1	2.2	8.4
Hordaland	516176	1.8	1.8	1.7	7.8
Møre og Romsdal	263086	2.0	2.0	2.0	8.7
Nordland	239510	1.8	1.8	1.8	7.0
Oppland	187193	1.8	1.8	1.9	6.8
Oslo	670085	1.4	1.4	1.3	7.7
Rogaland	469624	1.4	1.4	1.5	7.2
Sogn og Fjordane	107861	2.1	1.8	2.3	7.7
Telemark	170427	1.9	1.8	2.0	7,8
Troms	164591	1.6	1.5	1.7	6.2
Trøndelag	452329	2.1	2.2	2.1	10.0
Vest-Agder	184672	1.5	1.7	1.3	8.0
Vestfold	245950	1.8	1.7	1.8	7.3
Østfold	292307	1.7	1.7	1.7	7.3

## Discussion

This is the first nationwide study of glaucoma prevalence and incidence in Norway. The prevalence of glaucoma in the general Norwegian population in 2018 was 1,4%, rising to 8,0% in the population aged 70 years and older. Compared to previous statistics, we identified a higher prevalence [11]. This discrepancy may be explained by an aging population, improved transportation facilities, better access to ophthalmologists and opticians, improved diagnostic instruments such as ocular coherence tomography (OCT), and increased awareness of glaucoma subgroups, especially normal tension glaucoma.

The present study does not differentiate between different subtypes of glaucoma. The most prevalent types of glaucoma in Northern Europe are POAG and PEG, but we cannot isolate the relative proportions of these two open-angle glaucoma types in our material. The prevalence of angle closure glaucoma is low in Europe [16] and prophylactic iridotomy and early cataract extraction will likely keep the prevalence low, despite an aging population. A limitation of the study is persons with glaucoma that do not use anti-glaucomatous medication, due to early stage disease or in patients with previous laser or surgical treatment obviating the need for eye drops. These patients are not included in the study. However, we expect the number of these persons to be very low, because glaucoma is a progressive and, in most cases, bilateral disease. Discontinuation of medication after surgical treatment is often temporary. The bias related to patients receiving pressure-reducing eye drops due to temporary conditions (i.e. trauma, uveitis) were considered to be negligible. There is reasonable consensus regarding the definition of glaucoma, and the challenges linked to underdiagnosis and overtreatment discussed elsewhere [3, 4], apply also to our study. In Norway, all cases of glaucoma are diagnosed by an ophthalmologist, and following the introduction of automatic perimetry and OCT, the precision of diagnosis is high.

A Nordic study from 1989, estimated the glaucoma prevalence, based on drug consumption, in Norway to 0.92% [11]. However, only 6 Norwegian counties (representing 31.6% of the total population) were included, but interestingly, we found a similar trend towards higher or lower prevalence, compared to the national average, in these counties. A more recent study, comparable to our own, based on the Danish national prescription registry [12], showed a higher glaucoma prevalence in Denmark in 2012 (1.72%) than ours from 2018 (1.43%). These authors also reported increased prevalence in urban areas in Denmark. This is in contrast to our findings indicating marked rural variation, but not increased prevalence in urban areas. Our results are similar to other European reports using various methods and to larger meta-analytic studies [2, 5, 17–19]. Variation in glaucoma prevalence in Norway are to be expected as the occurrence of pseudoexfoliation syndrome varies geographically [20]. This seems to be applicable on a Scandinavian basis as the occurrence of capsular glaucoma in Denmark and southern Sweden is low [21, 22].

Several reports show increasing prevalence of glaucoma over time [2, 12, 23], some even predicting increased glaucomatous vision loss in the coming years [5, 24]. However, this presage is not supported in the present study, with our figures (Figs 4 and 7) indicating a slightly decreasing prevalence and incidence in the group 70 years and over. This is noteworthy because Norway has an ageing population, however, with immigration from European and non-European countries that may affect prevalence rates.

Estimated life-time prevalence was 9.4% for men and 10.2% for women in the present study. The higher life-time prevalence in women may be explained by the higher proportion of women with life expectancy over 90 years. Otherwise the prevalence rates were similar in all age groups for both genders.

The incidence of OAG varies considerably [13, 15]. In our nation-wide study glaucoma incidence proportion increased with age up to the 80–89 age group, followed by a decrease. One possible reason for this phenomenon could be that a large number of persons above 90 years are living in nursing homes. Pharmaceuticals supplied to these institutions are not covered by the “blue prescription system” which was the basis for our calculations. Another cause could be that many 90 year old people are multimorbid and have difficulty attending ophthalmological appointments.

In conclusion, the prevalence of glaucoma remained stable at 1.4% during the study period 2004–2018, whereas the incidence proportion fell slightly in patients above 70 years of age.

## Supporting information

S1 Data (XLSX)
